# N6‐isopentenyladenosine inhibits aerobic glycolysis in glioblastoma cells by targeting PKM2 expression and activity

**DOI:** 10.1002/2211-5463.13766

**Published:** 2024-03-21

**Authors:** Cristina Pagano, Laura Coppola, Giovanna Navarra, Giorgio Avilia, Beatrice Savarese, Giovanni Torelli, Sara Bruzzaniti, Erica Piemonte, Mario Galgani, Chiara Laezza, Maurizio Bifulco

**Affiliations:** ^1^ Department of Molecular Medicine and Medical Biotechnology University of Naples ‘Federico II’ Naples Italy; ^2^ Neurosurgery Unit A.O. San Giovanni di Dio e Ruggi d' Aragona – Salerno's School of Medicine Largo Città di Ippocrate Salerno Italy; ^3^ Institute of Endocrinology and Experimental Oncology (IEOS) National Research Council (CNR) Naples Italy

**Keywords:** cancer metabolism, glioblastoma, glycolysis, iPA, PKM2

## Abstract

Glioblastoma (GBM) is a primary tumor in the central nervous system with poor prognosis. It exhibits elevated glucose uptake and lactate production. This metabolic state of aerobic glycolysis is known as the Warburg effect. N6‐isopentenyladenosine (iPA), a natural cytokine modified with an isopentenyl moiety derived from the mevalonate pathway, has well‐established anti‐tumor activity. It inhibits cell proliferation in glioma cells, inducing cell death by apoptosis and/or necroptosis. In the present study, we found that iPA inhibits aerobic glycolysis in unmodified U87MG cells and in the same cell line engineered to over‐express wild‐type epidermal growth factor receptor (EGFR) or EGFR variant III (vIII), as well as in a primary GBM4 patient‐derived cell line. The detection of glycolysis showed that iPA treatment suppressed ATP and lactate production. We also evaluated the response of iPA treatment in normal human astrocyte primary cells, healthy counterpart cells of the brain. Aerobic glycolysis in treated normal human astrocyte cells did not show significant changes compared to GBM cells. To determine the mechanism of iPA action on aerobic glycolysis, we investigated the expression of certain enzymes involved in this metabolic pathway. We observed that iPA reduced the expression of pyruvate kinase M2 (PKM2), which plays a key role in the regulation of aerobic glycolysis, promoting tumor cell proliferation. The reduction of PKM2 expression is a result of the inhibition of the inhibitor of nuclear factor kappa‐B kinase subunit, beta/nuclear factor‐kappa B pathway upon iPA treatment. In conclusion, these experimental results show that iPA may inhibit aerobic glycolysis of GBM in stabilized cell lines and primary GBM cells by targeting the expression and activity of PKM2.

## Graphical abstract

The Warburg effect, also known as aerobic glycolysis, is characterized by high glucose uptake and lactate release even when sufficient oxygen is present. By reducing pyruvate kinase M2 (PKM2) via inhibition of the nuclear factor kappa B (NF‐κB) signaling pathway, N6‐isopentenyladenosine (iPA) decreases the Warburg effect in glioblastoma cells, thus impairing cancer cell proliferation.

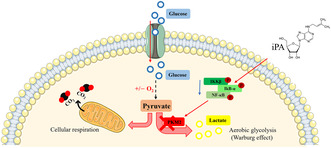

**Abbreviations**
ABM™: astrocyte basal medium
AGM™: astrocyte growth medium
DMEM: Dulbecco's modified Eagle's medium
ECAR: extracellular acidification rate
EGFR: epidermal growth factor receptor
EGFRvIII: U87MG‐EGFR‐vIII
GBM: glioblastoma
GLUT1: glucose transporter 1
HK1: hexokinase‐1
HK2: hexokinase‐2
IKKβ: inhibitor of nuclear factor kappa‐B kinase subunit, beta
iPA: N6‐isopentenyladenosine
IκB‐α: nuclear factor of kappa light polypeptide gene enhancer in B‐cells inhibitor, alpha
NHA: normal human astrocytes
NF‐κB: nuclear factor kappa B
PK: pyruvate kinase
PKM1: pyruvate kinase M1
PKM2: pyruvate kinase M2
RIPK1: receptor‐interacting protein kinase 1
RIPK3: receptor‐interacting protein kinase 3
U87/EGFR: EGFR‐overexpressed U87
U87MG‐EGFRwt: EGFR wild‐type
vIII: EGFR variant III

Glioblastoma (GBM) constitutes a highly aggressive brain tumor with a median survival rate between 12 and 24 months [1]. Histologically, the tumor is not only predominantly characterized by the presence of abnormal astrocytic cells, but also contains a mix of different cell types (including blood vessels) and areas of dead cells (necrosis) [2]. The current standard of care is surgical resection to the extent feasible, followed by adjuvant radiotherapy in combination with cycles of temozolomide (Stupp protocol) [3]. However, GBM patients develop resistance to therapy resulting in tumor recurrence. The major determinant of therapeutic resistance is a result of intra‐ and intertumoral heterogeneity and the presence in GBM of a subpopulation of self‐renewing and pluripotent GBM stem‐like cells responsible for GBM invasiveness and recurrence [4, 5]. GBM are characterized by a complicated genetic profile and high variability genetic, forcing a reconsideration of traditional therapy [6]. In this scenario, the development of new drugs aiming to improve overall survival time and quality of life for these patients is important. GBMs have a high frequency in specific molecular alteration, such as amplification and mutation of the epidermal growth factor receptor (EGFR) gene, encountered in up to 60% of GBMs, and especially the truncation of the EGFR protein extracellular domain, EGFR variant III (vIII) that promotes proliferation, angiogenesis, invasion and resistance to therapy [7, 8]. Moreover, GBM cells up‐regulate glycolysis, a phenomenon known as the Warburg effect [9, 10]. In this metabolic state, the glycolytic pathway is increased to quickly produce ATP per mole of glucose, ensuring the energy required by highly proliferating cancer cells, particularly in a hypoxic environment. However, several studies have found that GBM cells exhibit a vast variability in mitochondrial respiration and glucose dependency [11]. A key enzyme in the regulation of cell metabolism is pyruvate kinase isoenzyme (PK). It catalyzes the conversion of phosphoenolpyruvate to pyruvate and ATP. The most studied isomeric forms of PK are: PK type M1 (PKM1) and PK type M2 (PKM2). They have distinct expression patterns and functions. PKM2 is expressed in highly proliferating tissues and is overexpressed in several types of cancer, including GBM, whereas PKM1 is expressed in normal differentiated tissues [12, 13, 14]. The elevated expression and activity of PKM2 is essential for the Warburg effect, promoting the switch of the cell metabolism to aerobic glycolysis [15]. Recently, it was reported that activation of EGFR in glioma results in elevated glucose uptake and lactate production, and the PKM2 levels correlated with levels of EGFR activity in GBM. Moreover, PKM2 upregulation is dependent to EGFR‐induced nuclear factor kappa B (NF‐κB) activation during tumor progression [16]. N6‐isopentenyladenosine (iPA), a natural cytokinin consisting of adenosine and an isopentenyl moiety, has pleiotropic properties that are able to inhibit the growth of several cancer cells through several mechanisms of action, including the arrest of the cell cycle and the induction of apoptosis *in vitro* and *in vivo*. Recently, we observed that it inhibited the migration, angiogenic process and vasculogenic mimicry of stabilized GBM cell lines and patient‐derived GBM cells [17, 18, 19, 20, 21, 22]. In another study, we demonstrated that iPA treatment induced necroptosis in GBM cells through the activation of receptor‐interacting protein kinase 1 (RIPK1), receptor‐interacting protein kinase 3 (RIPK3) and mixed lineage kinase domain‐like protein, allowing the necrosome formation and the execution of necroptotic process [23]. Lastly, we have reported that IPA treatment inhibited mitochondrial respiration in U87MG cells and the same cells engineered to over‐express EGFR wild‐type (U87MG‐EGFRwt) or EGFRvIII (U87MG‐EGFR‐vIII), as well as in primary GBM patients‐derived cells, by preventing the translocation of EGFR/EGFRvIII on the mitochondria through the inhibition of Y845 phosphorylation of EGFR [24]. In the present study, we observed that this natural compound inhibited aerobic glycolysis in the GBM cells described above by targeting PKM2 expression.

## Materials and methods

### Drugs and reagents

For all of the experiments, the solutions were prepared starting from stock solution. iPA was purchased from Sigma‐Aldrich (St Louis, MO, USA), solubilized in dimethyl sulfoxide from a starting concentration of 10 mm, and added to cell cultures growth medium in increasing concentrations (0–10 μm) depending on the experiment. DASA‐58 was purchased from Sigma‐Aldrich (SML2853) and added to cell cultures growth medium at the final concentration of 20 μm. For each experiment, fresh dilutions were made from the stock solution and added to cell cultures in supplemented medium.

### Cell culture

The GBM cell lines used for the present study are U87MG, purchased from Elabscience (Elabscience, Houston, TX, USA; catalog no. EP‐CL‐0238), as well as U87MG expressing EGFRwt and U87MG expressing EGFRvIII were kindly donated by F. B. Furnari of the Ludwig Institute for Cancer Research and the Moores Cancer Center (University of California, San Diego, La Jolla, CA, USA) [25]. All stabilized cell lines were cultured in Dulbecco's modified Eagle's medium (DMEM) (Gibco, Thermo Fisher Scientific, Monza, Italy) supplemented with 10% heat inactivated fetal bovine serum, 1% l‐glutamine, 1% sodium pyruvate, 1% non‐essential amino acids (Lonza, Rome, Italy) and 0.1% Plasmocin™ (InvivoGen, San Diego, CA, USA).

Normal human astrocytes (NHA), purchased from Life Sciences (Rome, Italy; Product Code: P10251), were grown in astrocyte basal medium (ABM™) supplemented with astrocyte growth medium (AGM™) SingleQuots Kit (Lonza). All cell cultures were maintained at 37 °C in humidified 5% CO_2_ atmosphere.

For primary cell line (here designed as GBM4), the patients underwent tumor resection at the Neurosurgery Service of ‘Antonio Cardarelli’ Medical Hospital (Napoli, Italy). All tissue samples were collected in accordance with the ethical standards of the Institutional Committee (DEL. N°897, August 13, 2020). Informed consent in written form was obtained from all subjects involved in the study. The samples of brain tissue containing the tumor were processed immediately to obtain cultured cells in accordance with the previously described protocol [21].

For each tumor sample, Illumina EPIC ARRAY 850 Beads‐Chip (850K) (Illumina, San Diego, CA, USA) was used to evaluate the DNA methylation status of 850 000 CpG sites, in accordance with the manufacturer's instructions, as previously described[20]. Similarly, for each tumor sample, IDH1/IDH2 mutational status and MGMT methylation assessment were conducted as previously described [20].

### Ethical approval and consent to participate

The study was conducted according to the guidelines of the Declaration of Helsinki and approved by the Ethics Committee of Neurosurgery Service of ‘Antonio Cardarelli’ Medical Hospital (Napoli, Italy) (DEL. N°897, August 13, 2020). Informed consent was obtained from all subjects involved in the study. Written informed consent was obtained from the patients to publish this paper.

### Western blot analysis

Total protein lysates were extracted using RIPA lysis buffer (50 mm Tris‐HCl, 150 mm NaCl, 0.5% Triton X‐100, 0.5% deoxycholic acid, 10 mg·mL^−1^ leupeptin, 2 mm phenylmethylsulfonyl fluoride and 10 mg·mL^−1^ aprotinin; Sigma‐Aldrich) supplemented with phosphatase and protease inhibitors. Equal amounts of lysate were resolved on SDS/PAGE gel and transferred by blotting onto nitrocellulose membranes. Upon saturation, membranes were incubated with primary antibodies to probe the proteins of interest and then probed with appropriate horseradish peroxidase‐conjugated secondary antibodies. Quantitative estimation was performed by measuring densitometric band intensity using imagej (NIH, Bethesda, MD, USA). For western blot analysis, the antibodies used were: rabbit monoclonal anti‐PKM2 (Cell Signaling Technology, Denvers, MA, USA; #4053), rabbit polyclonal anti‐IκB‐α (Santa Cruz Biotechnology, Inc., Dallas, TX, USA; sc‐371), rabbit polyclonal anti‐p‐IκB‐α (Ser32/S36) (Elabscience; E‐AB‐20911), rabbit polyclonal anti‐IKKβ (Cell Signaling Technology; #2678), rabbit polyclonal anti‐phospho‐IKKα/β (S176/180) (Elabscience; E‐AB‐68178), mouse monoclonal anti‐ NF‐κB (Cell Signaling Technology; #6956), mouse monoclonal anti‐phospho‐ NF‐κB (Ser536) (Cell Signaling Technology; #13346), mouse monoclonal anti‐β‐actin (Santa Cruz Biotechnology, Inc.; sc‐47778) and rabbit polyclonal anti‐α‐tubulin (Cell Signaling Technology; #2144).

### Seahorse analysis

Metabolic profile was evaluated in GBM stabilized cell lines (U87MG, U87MG‐EGFRwt and U87MG‐EGFRvIII), as well as NHA and GBM primary cell line (designed as GBM4), treated or not with iPA for 18 and 24 h. Real‐time measurements of extracellular acidification rate (ECAR) were performed using an XFe‐96 Analyzer (Agilent Technologies, Santa Clara, CA, USA), as previously reported with some adjustments [26]. Specifically, cells were plated in XFe‐96 plates (Agilent Technologies) at a concentration of 2 × 10^4^ cells/well and cultured with DMEM medium. ECAR was measured in XF DMEM medium (Agilent Technologies) in a basal condition and in response to 10 mm glucose, 5 μm oligomycin and 100 mm of 2DG (all from Sigma‐Aldrich). Experiments with the Seahorse were performed with the assay conditions: 3‐min mixture, 3‐min wait and 3‐min measurement. Parameters of the glycolytic pathway were calculated from the ECAR profile: glycolysis was calculated as the difference between ECAR values before oligomycin injection and ECAR values before glucose injection; glycolytic capacity was calculated as the difference between ECAR values after oligomycin injection and ECAR values before glucose injection; and glycolytic reserve was calculated as the difference between glycolytic capacity and glycolysis.

### ATP assay

The CellTiter‐Glo^®^Luminescent Cell Viability Assay kit (Promega Italia s.r.l, Milano, Italy; product code: G7570) was used to evaluate the ATP levels, in accordance with the manufacturer's instructions. For the execution of this assay, 2.5 × 10^4^ cells were seeded in 96‐well plates. Briefly, after equilibrating the plate at room temperature for approximately 30 min, 100 μL of CellTiter‐Glo^®^ Reagent were added per well. After mixing the content for approximately 2 min to allow cell lysis, the plate was incubated at room temperature for 10 min to stabilize the luminescent signal, which was subsequently evaluated by BioTek's Synergy™ HT Luminometer (BioTek Instruments Inc., Winooski, VT, USA).

### BrdU assay

For the BrdU assay, the experiment was performed using a 5‐bromo‐2′‐deoxyuridine ELISA kit (Roche, Basel, Switzerland) in accordance with the manufacturer's instructions. GBM cells were seeded in a flat bottom 96‐well plates at a density of 1 × 10^4^ cells/well in 100 μL of culture medium for 24 h. Cells were then incubated with iPA at the indicated concentrations (from 0 to 10 μm) for 48 h. Subsequently, the cells were incubated for about 2 h with 10 μL of BrdU/well, added to the medium at a concentration of 100 μm (BrdU Labeling Solution diluted 1 : 100 in sterile medium). Culture medium was then removed and 100 μL of FixDenant included in the kit was added per well. After 30 min of incubation, the FixDenant solution was removed. Cells were then incubated for approximately 90 min with 100 μL/well of anti‐BrdU‐POD diluted in accordance with the instructions. After the cells were washed once with phosphate‐buffered saline to remove the unbound antibody, substrate solution (100 μL/well) was added. The absorbance values were measured at a wavelength of 450 nm using a Synergy HT Microplate Reader (BioTek Instruments Inc.).

### RNA isolation and quantitative real‐time PCR

Total RNA extraction procedures were performed using EuroGold Trifast reagent (EuroClone, Pavia, Italy; product code: EMR507100) in accordance with the manufacturer's instructions. Before extraction, cells were seeded on p60 dishes at a density of 8 × 10^5^ cells·cm^−2^ in supplemented DMEM medium. The obtained RNA samples were measured using a NanoDrop spectrophotometer (Thermo Fisher Scientific). Five hundred nanogram of purified RNA were reverse transcribed into cDNA using SuperScript II Reverse Transcriptase (Invitrogen, Carlsbad, CA, USA). Quantitative real‐time PCR analysis was performed using gene‐specific primers and a SYBR Green I (Promega Italia s.r.l) fluorescent dye. Quantitative real‐time PCR reactions were run in triplicate using the CFX Opus 96 Real‐Time PCR System (Bio‐Rad Laboratories GmbH, Munich, Germany) with the cycling conditions: initial denaturation step at 95 °C for 5 min, followed by 44 cycles (95 °C for 15 s, 60 °C for 1 min). Data were analyzed using cfx manager, version 3.0 (Bio‐Rad Laboratories GmbH) and a relative quantification of gene expression was determined using the ∆∆Ct method. β‐2‐microglobulin mRNA was used as an endogenous control. All primer sequences are reported in Table 1.

**Table 1:** Primers used for quantitative PCR analyses. GLUT1, glucose transporter 1; HK1, hexokinase 1; HK2, hexokinase 2; PKM2, pyruvate kinase M2.

Primer ID	Forward (5′ to 3′)	Reverse (5′ to 3′)
GLUT1	5′‐CACCACCTCACTCCTGTTACTT‐3′	5′‐TCTCACTCCCATCCAAACCTC‐3′
HK1	5′‐GGACTGGACCGTCTGAATGT‐3′	5′‐ACAGTTCCTTCACCGTCTGG‐3′
HK2	5′‐CAAAGTGACAGTGGGTGTGG‐3′	5′‐GCCAGGTCCTTCACTGTCTC‐3′
PKM2	5′‐GAACATCCTGTGGCTGGACT‐3′	5′‐GCACCTTTCTGCTTCACCTG‐3′
β‐2‐microglobulin	5′‐CCT GAA TTG CTA TGT GTC TGG G‐3′	5′‐ACA CGG CAG GCA TAC TCA TC‐3′

### Lactic acid assay kit

a l‐lactic acid Colorimetric Assay Kit (Elabscience; Cat.No.: E‐BC‐K044‐S) was used to assess lactate content in samples. This colorimetric assay was performed in triplicate in a 96‐well plate, whereas the absorbance, at the specific wavelength of 530 nm, was measured using a BioTek Synergy™ HT Luminometer (BioTek Instruments Inc.).

### Statistical analysis

Statistical analysis was performed using prism, version 9.0 (GraphPad Software Inc., San Diego, CA, USA). The data are reported as the mean ± SD and analyzed for statistical significance using a two‐tailed Student's *t*‐test for independent groups or by one/two‐way ANOVA followed by Bonferroni correction for multiple comparisons. *P* < 0.05 was considered statistically significant.

## Results

### iPA suppresses aerobic glycolysis of GBM cells

**Fig. 1:**
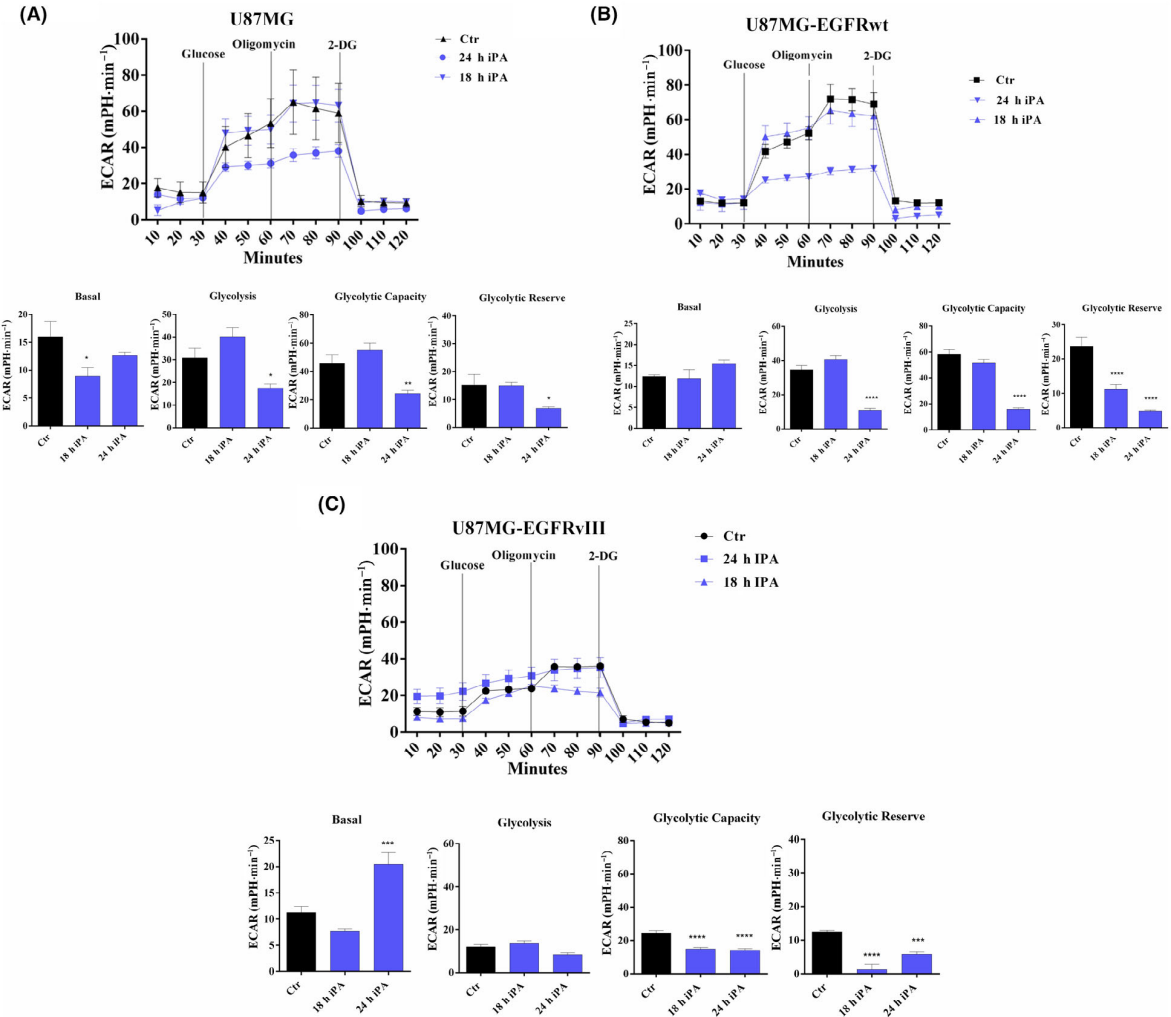
iPA suppresses aerobic glycolysis of GBM cells. Seahorse analysis showed a reduction in ECAR parameters after 24 h of iPA 10 μm treatment. Upper: kinetic profile of ECAR of (A) U87MG, (B) U87MG‐EGFRwt and (C) U87MG‐EGFRvIII cells, treated or not with iPA for 18 and 24 h. Lower: parameters of the glycolytic pathway. Data are from three independent experiments in technical replicate. Data are expressed as the mean ± SEM. *P < 0.05, **P < 0.01, ***P < 0.001, ****P < 0.0001 (Student's *t*‐test and one/two‐way ANOVA).

**Fig. 2:**
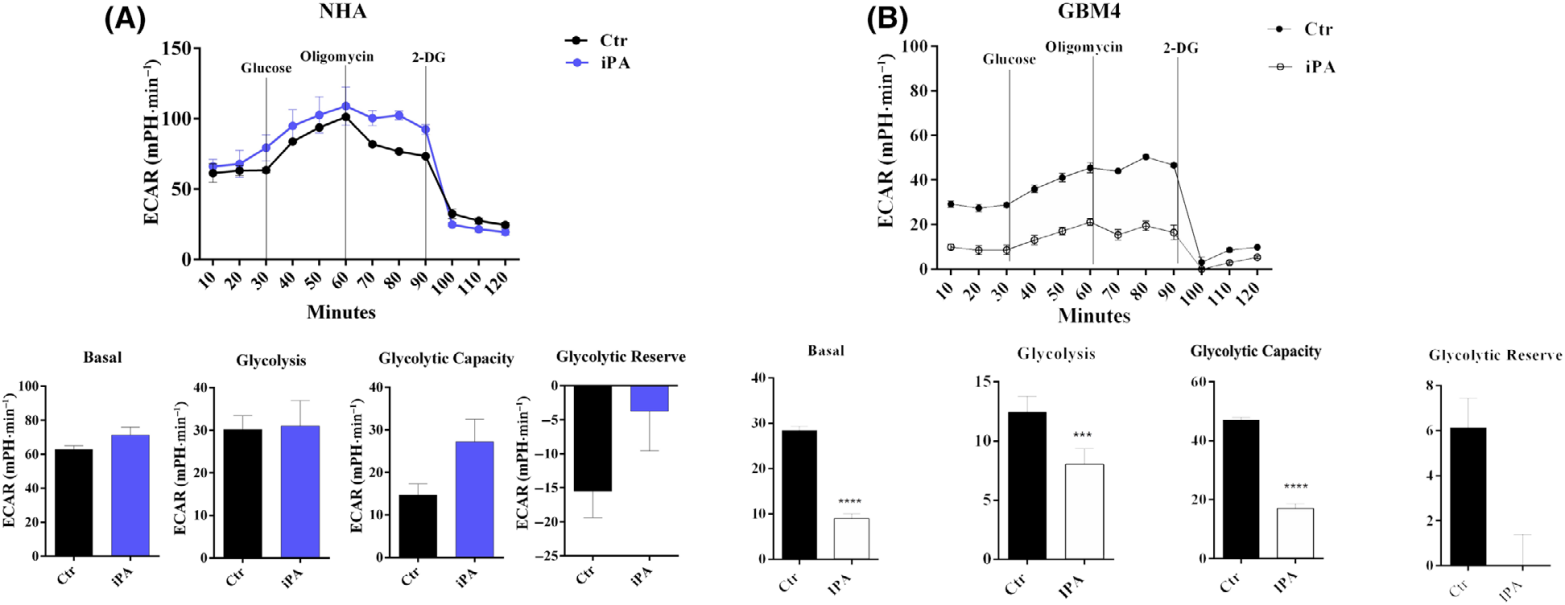
iPA reduces aerobic glycolysis of GBM primary cell line but elicits no effects in NHA cells. Seahorse analysis showed that iPA 10 μm decreases ECAR in GBM4 cell line after 24 h of treatment. The same treatment showed no effects in the GBM normal counterparts, NHA cells. Upper: kinetic profile of ECAR of (A) NHA cells and (B) GBM4, treated or not with iPA for 24 h. Lower: parameters of the glycolytic pathway. Data are from three independent experiments in technical replicate. Data are expressed as the mean ± SEM. ***P < 0.001, ****P < 0.0001 (Student's *t*‐test and one/two‐way ANOVA).

**Fig. 3:**
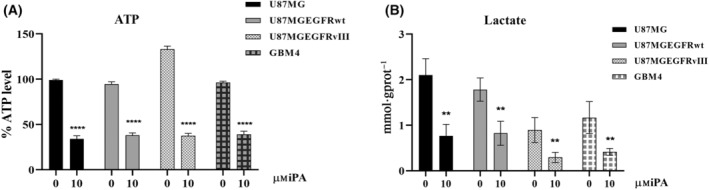
iPA reduces ATP and lactate production in GBM cells. (A) Schematic representation of the measurement of cellular ATP content in U87MG, U87MG‐EGFRwt, U87MG‐EGFRvIII and GBM4 cells. Twenty‐four hours of iPA 10 μm reduced ATP production in all cell lines treated. (B) Schematic representation of lactate production in U87MG, U87MG‐EGFRwt, U87MG‐EGFRvIII and GBM4 cells. iPA 10 μm reduced these parameters, thus impairing glycolytic activity in GBM cell lines. All data are presented as the mean ± SD of at least three independent experiments. **P < 0.01, ****P < 0.0001 (Student's *t*‐test and one/two‐way ANOVA).

**Fig. 4:**
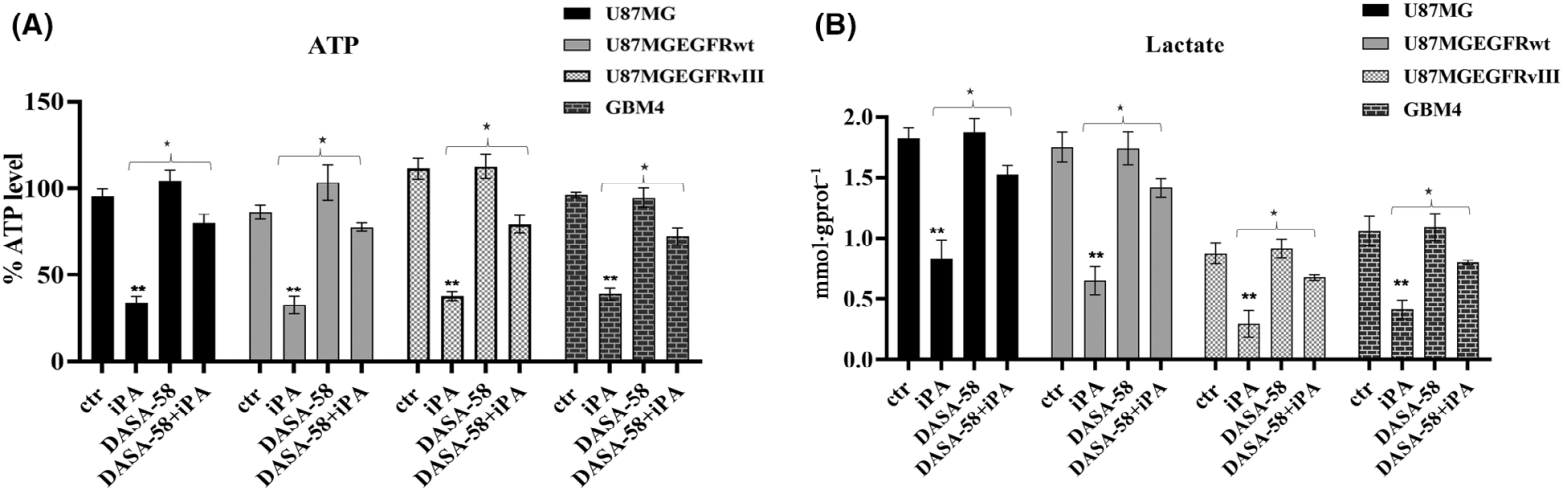
iPA effects on ATP and lactate production is rescued by DASA‐58. (A) Schematic representation of the measurement of cellular ATP content in U87MG, U87MG‐EGFRwt, U87MG‐EGFRvIII and GBM4 cells. When pre‐treated with DASA‐58 20 μm for 12 h, ATP production of GBM cells was not reduced by 24 h of iPA 10 μm treatment. (B) Schematic representation of lactate production in U87MG, U87MG‐EGFRwt, U87MG‐EGFRvIII and GBM4 cells. When pre‐treated with DASA‐58 20 μm, lactate production of GBM cells was not reduced by iPA 10 μm treatment. All data are presented as the mean ± SD of at least three independent experiments. *P < 0.05, **P < 0.01 (Student's *t*‐test and one/two‐way ANOVA).

Recently [23], we observed that growing concentrations of iPA ranging from 0 to 10 μm reduced the proliferation in U87MG, in U87MG‐EGFRwt, in U87MG‐EGFRvIII and in primary cell line GBM4 after 48 h of treatment, whereas no significant effect was showed in the NHA (Fig. S1A). Furthermore, as shown in Fig. S1B, 0–10 μm iPA showed no significant effect on cell viability at 24 h of treatment (Fig. S1B). iPA treatment at 10 μm for 48 h caused necroptosis in GBM cells lines and GBM4 primary cell isolated by cancer biopsy through the activation of necroptosis markers, whereas, in treated NHA cells, apoptotic events were irrelevant. Moreover, in a recent study, we demonstrated that iPA interferes with mitochondrial bioenergetic capacity in the same cells as those used in the present study [24]. Given that aerobic glycolysis has a key role for most tumor cells in the maintainance of rapid growth and in metastasis, we tested whether IPA inhibited tumor cell growth via blocking the aerobic glycolysis in GBM cells. To verify this hypothesis, the ECAR was assessed using a Seahorse Extracellular Flux XFe‐96 Analyzer in stabilized GBM cell lines treated with iPA 10 μm for 24 h. The results shown in Fig. 1 indicate that iPA treatment significantly decreased ECAR parameters including the glycolytic activity and capacity compared to the vehicle‐treated control group, indicating inhibition of the aerobic glycolysis (Fig. 1A–C). To obtain further insight into this mechanism, we have also evaluated the response of iPA treatment in NHA primary cells. The ECAR analysis performed on NHA cells resulted in no significant changes in the aerobic glycolysis as shown in Fig. 2A. We also confirmed the reduction of ECAR parameters in GBM4, a primary culture, as shown in Fig. 2B. Moreover, to examine whether the glycolytic activity was inhibited following iPA administration, lactate and ATP production were determined (Fig. 3). We observed a reduction of ATP (U87MG, 35 ± 0.8%; U87MG‐EGFRwt, 37 ± 0.8%; U87MG‐EGFRvIII, 38 ±0.75%; GBM4, 40 ± 0.8%, compared to the untreated group) (Fig. 3A) and lactate production (U87MG, 0.75 ± 1.8 mmol g protein^−1^; U87MG‐EGFRwt, 0.80 ± 2 mmol g protein^−1^; U87MG‐EGFRvIII, 0.35 ± 0.5 mmol g protein^−1^; GBM4, 0.45 ± 0.25 mmol g protein^−1^, compared to the untreated group) (Fig. 3B), suggesting that the suppressed glycolytic activity occurred at an intracellular level. Because the PKM2 is overexpressed in GBM [12, 13, 14, 15] and its elevated expression and activity are essential for the Warburg effect, we have explored the functional relevance of PKM2 in GBM cells. To this end, we have analyzed the effect of specific small‐molecule PKM2 activator, DASA‐58 [27], in GBM cells treated with iPA 10 μm. DASA‐58 activates PKM2 binding to the dimer–dimer interface of PKM2 to permit its tetramerization. We pre‐treated the GBM cells with PKM2 agonist DASA‐58 for 12 h; we then added iPA for another 24 h, afterwards ATP and lactate production was determined. We observed that the parameters analyzed were partly reversed, suggesting that iPA treatment affects PKM2 enzymatic activity. In particular, we observed an increase of approximately 50% in ATP and lactate production in the GBM cells treated with DASA‐58 + iPA compared to those treated with iPA alone (Fig. 4A,B).

### iPA reduces the expression of PKM2

**Fig. 5:**
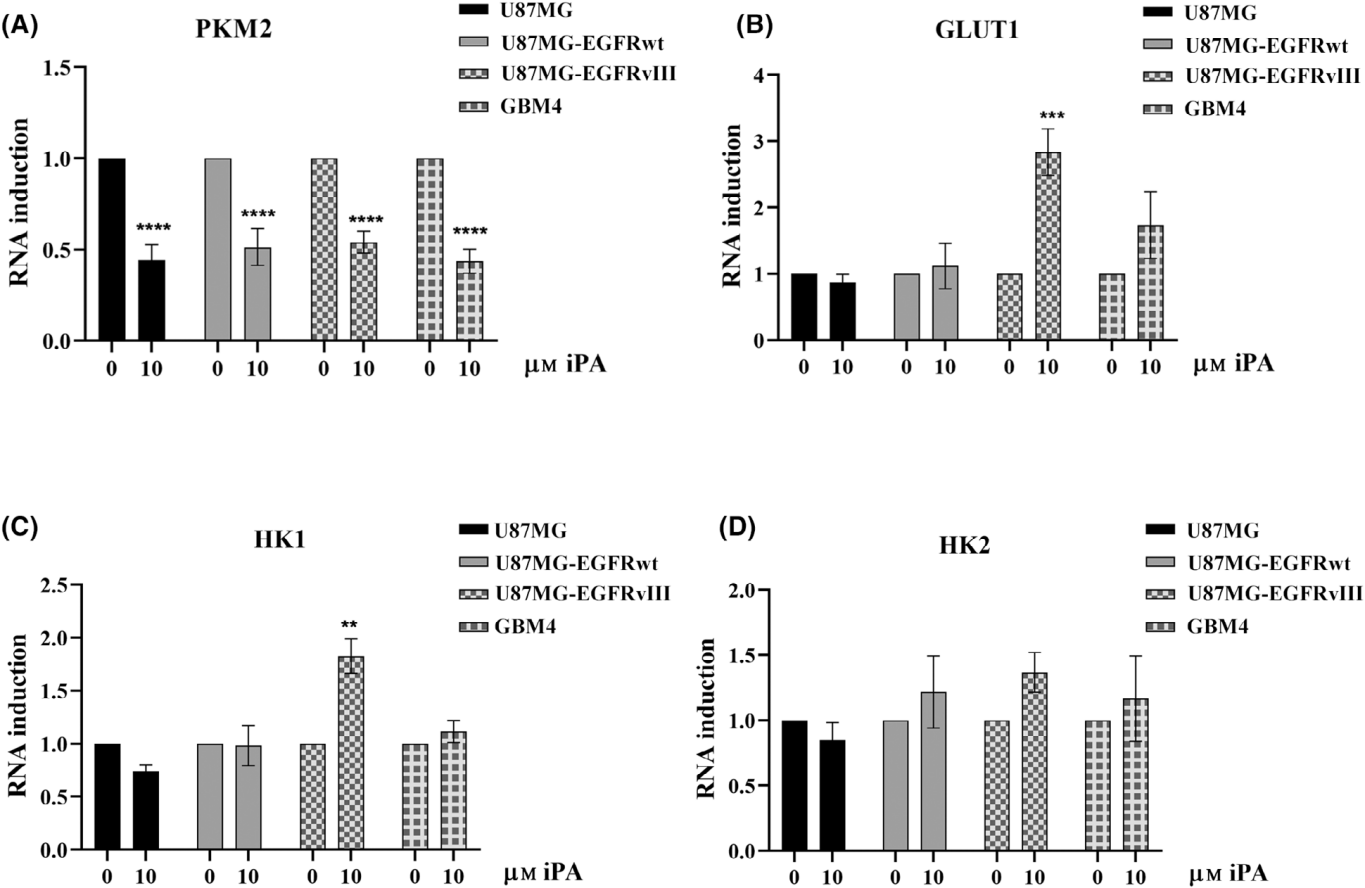
iPA modulates the expression of key rate‐limiting enzymes. Real‐time PCR analysis of expression of genes belonging to the glycolytic pathway. (A) After 24 h, iPA 10 μm reduced the expression of PKM2 enzyme, a critical regulator of the Warburg effect. (B–D) iPA effects on GLUT1, HK1 and HK2 expression were different depending on the cell line analyzed. All data are presented as the mean ± SD of at least three independent experiments. **P < 0.01, ***P < 0.001, ****P < 0.0001 (Student's *t*‐test and one/two‐way ANOVA).

To further investigate how iPA inhibits aerobic glycolysis, we explored the effects of this drug on the expression of four rate‐limiting enzymes: glucose transporter 1 (GLUT1), hexokinase‐1 (HK1), hexokinase‐2 (HK2) and PKM2 of the glycolysis pathway. We observed that iPA at 10 μm after 24 h attenuated the transcript levels of PKM2 (Fig. 5A) in U87MG, as well as in U87MG‐EGFRwt and U87MG‐EGFRvIII and primary GBM4 cells. The expression of GLUT1, HK1 and HK2 remained unchanged in U87MG, U87MG‐EGFRwt and GBM4, whereas, in U87MG‐EGFRvIII, GLUT1 and HK1 levels were increased (Fig. 5B–D). Although these data could prove to be interesting in the future, we decided to focus our analysis on the modulation of PKM2. PKM2 has been shown to play a critical role in switching tumor cell metabolism from oxidative phosphorylation to aerobic glycolysis, contributing to GBM genesis.

### iPA suppresses PKM2 expression by regulating the NF‐κB signal pathway

**Fig. 6:**
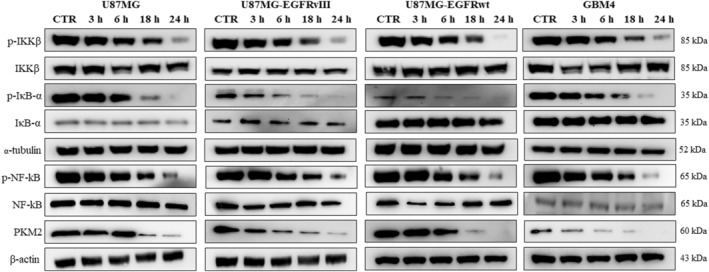
iPA suppresses PKM2 expression through the inhibition of NF‐κB signal pathway. Representative images of western blot analysis of key proteins belonging to the NF‐κB signal pathway and of PKM2. Levels of phosphorylated proteins and of PKM2 were reduced after 24 h of iPA 10 μm treatment. Densitometry analysis is showed in Figs [Link], [Link]. Densitometry analysis is presented as the mean ± SD of at least three independent experiments.

Recently, it was demonstrated that EGFR activation in human cancer cells results in increased glucose uptake and lactate production in a PKM2 expression dependent manner. Furthermore, EGF induced PKM2 upregulation is dependent on activation of a PLCγ1‐PKCε‐IKKβ‐RelA signaling cascade [16]. Thus, considering that higher levels of PKM2 expression correlate with an elevated activity of EGFR and inhibitor of NF‐kB kinase subunit beta (IKKβ)‐dependent pathways in human GBM cell lines, we decided to investigate iPA effects on the phosphorylation state of IKKβ‐NF‐kβ pathway in U87MG, U87MG‐EGFRwt, U87MG‐EGFRvIII and GBM4 primary cells. NF‐κB is the heterodimer of the p50 and p65 subunits that is responsible for transcriptional activity [28]. Employing western blot analysis, we tested the effect of iPA at 10 μm for 3, 6, 18 and 24 h on the levels of constitutively expressed NF‐κB/p65 nuclear protein in GBM cells. In these cells, iPA did not cause a decrease in NF‐κB/p65 protein levels (Fig. 6), whereas we observed inhibition of phosphorylation of NF‐κB in cells treated with iPA at 18 h, compared to the control group. Then, we analyzed the phosphorylation state of nuclear factor of kappa light polypeptide gene enhancer in B‐cells inhibitor, alpha (IκB‐α), which is a prerequisite for the entry of NF‐κB into the nucleus and its subsequent activation [28]. As shown in Fig. 6, we observed the reduction of phosphorylation of IκB‐α following iPA treatment (Figs [Link], [Link]). Furthermore, because NF‐κB is activated by IKKβ [25], which phosphorylates and thereby initiates signals required for degradation of IκB‐α, a negative NF‐κB regulator, we examined the iPA effect on IKKβ phosphorylation. We observed that iPA significantly decreased the phosphorylation of IKKβ after 18 h of treatment. These results suggest that iPA treatment interferes with the NF‐κB pathway.

## Discussion

In the present study, we have demonstrated that iPA treatment at low micromolar concentrations (10 μm) inhibited aerobic glycolysis of stabilized GBM cells lines, as well a primary cell line (GBM4), reducing PKM2 expression. Indeed, data from the ECAR analysis indicated a strong reduction of the glycolysis and the glycolytic capacity after 24 h of iPA treatment. To obtain further insight into this mechanism, we also performed ECAR analysis in NHA primary cells treated with iPA at 10 μm. We did not observe significant changes in the aerobic glycolysis in treated NHA cells compared to the control groups. Metabolites linked to glycolytic activity such as ATP and lactate production [29] were reduced in GBM cells upon iPA treatment for 24 h compared to control cells. These results suggest that the suppression of growth of GBM cells induced by iPA is a result of inhibition of the glycolytic activity. Indeed, in our previously published data, we observed that iPA treatment of GBM cells for 48 h at 10 μm exerts anti‐tumor effects by inducing apoptosis and necroptosis [21, 23]. Here, to examine the mechanism of iPA action on the glycolytic pathway, we analyzed the expression of some enzyme involved in this pathway in the GBM cells. We observed that, in all GBM cells treated with iPA, the PKM2 enzyme expression and the protein levels were reduced. To corroborate these data, we analyzed the effect of specific small‐molecule PKM2 activator, DASA‐58 [30], in GBM cells treated with iPA 10 μm. We noted that the pre‐treatment with DASA‐58 partly reversed the inhibitory effect of iPA on the ATP and lactate production, suggesting that the target of iPA action was the PKM2 enzyme. This enzyme regulates the glycolytic intermediates production playing a crucial role in cell energy supply [31]. Some studies have reported that PKM2 is overexpressed in glioma compared to control, while glioma‐adjacent tissues only showed a slight PKM2 overexpression, suggesting that PKM2 overexpression could be an important trigger for glioma progression [32]. Other studies have reported that the EGFR activation determined the nuclear translocation of PKM2, but not PKM1. In addition, U87MG overexpressing EGFRvIII mutant had a higher amount of nuclear PKM2 than U87MG. In the nucleus, PKM2 binds to c‐Src‐phosphorylated Y333 of β‐catenin, promoting cancer cells proliferation [33]. Moreover, EGFR activation caused a direct interaction between PKM2 and histone H3, favoring cell proliferation and brain tumorigenesis [34]. Recently, it was observed that EGFR activation results in PKM2 upregulation dependent on the IKKβ‐ NF‐κB pathway [16]. Another study demonstrated that fenofibrate reduced aerobic glycolysis through the inhibition of the transcriptional activity of NF‐κB/RelA and its association with hypoxia inducible factor1 alpha required for the binding of NF‐κB/RelA to the PKM promoter [35]. Because, in our previous studies, we demonstrated that iPA treatment affected the activity of IKKβ‐NF‐κB pathway [36], the present study tested the phosphorylation state of IKKβ/NFKB pathway in GBM cells treated with iPA. We observed that iPA treatment of GBM cells inhibited the phosphorylation of IKKβ, IκBα and NF‐κB, thus preventing the NF‐κB interaction with PKM promoter causing the reduction of PKM2 expression and proteins levels (Figs 5 and 6). Finally, the expression of GLUT1, HK1 and HK2 was also detected in the present study. HK is an enzyme that regulates the first step of glycolytic pathway. Several studies have confirmed that GLUT1, HK1 and HK2 expression is closely related to the progression of malignant tumors [37]. We observed that these enzymes did not vary significantly in GBM cells. More, we observed that the expression of GLUT1 and HK1, which phosphorylates glucose to glucose 6‐phosphate, the first rate‐limiting step in glycolysis [38], was increased in U87MG‐EGFRvIII when treated with iPA. Currently, we cannot explain these data; however, it may be a compensatory mechanism implemented by cells to respond to the lack of energy support. These results demonstrate that iPA inhibits tumor growth by inhibiting PKM2‐mediated aerobic glycolysis in GBM cells, suggesting the potential for using iPA in therapeutic developments. Currently, PKM2 is considered as a potential therapeutic target for cancer treatment, promoting the research of PKM2 inhibitors [39]. Several studies have shown that inhibition of PKM2 expression by specific short hairpin RNA and microRNA leads to the death of cancer cells, decreasing metabolic activity and reducing tumorigenesis. Moreover, many small molecule inhibitors and hormones are able to inhibit cell proliferation by targeting PKM2, leading to the reduction of aerobic glycolysis in cancer cells [40]. In the present study, we found that iPA inhibits the expression and protein levels of PKM2, failing to provide energy and metabolic intermediates for the growth of GBM cells. These data suggest the potential for using iPA in target therapy; however, more studies are necessary to evaluate the efficacy and safety of iPA in the treatment of GBM.

## Conflicts of interest

The authors declare that they have no conflicts of interest.

## Author contributions

CP and CL designed the study and researched data. GN, LC, GA and BS performed the majority of the experiments. SB, EP helped with experiments. GT managed patients and performed tumor samples collection. CL and CP wrote the manuscript. MB and MG contributed to the discussion and reviewed the manuscript. All authors approved the final version of the manuscript submitted for publication.

## Supporting information


**Fig. S1.** iPA effects on GBM cells proliferation and viability. (A) BrdU assay showing the effects of scalar doses of iPA (1–10 μm) after 48 h on NHA and GBM cell lines. Notably, iPA did not elicit any effects on the proliferation of NHA cells, the normal counterpart of GBM cells. (B) MTT assay showing that iPA (1–10 μm) after 24 h had no significant effects on GBM cells and NHA viability. All data are presented as the mean ± SD of at least three independent experiments. ****P < 0.0001 (Student's *t*‐test and one/two‐way ANOVA).

**Fig. S2.** Western blot densitometry analysis of U87MG. Histograms showing densitometry analysis of p‐IKKβ/IKKβ, p‐IkBα/IkBα, p‐NF‐kB/NF‐kB and PKM2/β‐actin bands of the U87MG cell line. All data are presented as the mean ± SD of at least three independent experiments. ****P < 0.0001 (Student's t‐test and one/two‐way ANOVA).

**Fig. S3.** Western blot densitometry analysis of U87MG‐EGFRwt. Histograms showing densitometry analysis of p‐IKKβ/IKKβ, p‐IkBα/IkBα, p‐NF‐kB/NF‐kB and PKM2/β‐actin bands of the U87MG‐EGFRwt cell line. All data are presented as the mean ± SD of at least three independent experiments. ****P < 0.0001 (Student's *t*‐test and one/two‐way ANOVA).

**Fig. S4.** Western blot densitometry analysis of U87MG‐EGFRvIII. Histograms showing densitometry analysis of p‐IKKβ/IKKβ, p‐IkBα/IkBα, p‐NF‐kB/NF‐kB and PKM2/β‐actin bands of the U87MG‐EGFRvIII cell line. All data are presented as the mean ± SD of at least three independent experiments. ****P < 0.0001 (Student's *t*‐test and one/two‐way ANOVA).

**Fig. S5.** Western blot densitometry analysis of GBM4. Histograms showing densitometry analysis of p‐IKKβ/IKKβ, p‐IkBα/IkBα, p‐NF‐kB/NF‐kB and PKM2/β‐actin bands of the GBM4 cell line. All data are presented as the mean ± SD of at least three independent experiments. ****P < 0.0001 (Student's t‐test and one/two‐way ANOVA).

## Data Availability

The data presented in the study are available from the corresponding author upon reasonnable request.

## References

[1] Cloughesy TF , Cavenee WK and Mischel PS (2014) Glioblastoma: from molecular pathology to targeted treatment. Annu Rev Pathol 9, 1–25. 23937436 10.1146/annurev-pathol-011110-130324

[2] Eder K and Kalman B (2014) Molecular heterogeneity of glioblastoma and its clinical relevance. Pathol Oncol Res 20, 777–787. 25156108 10.1007/s12253-014-9833-3

[3] Stupp R , Mason WP , van den Bent MJ , Weller M , Fisher B , Taphoorn MJ , Belanger K , Brandes AA , Marosi C , Bogdahn U *et al*. (2005) Radiotherapy plus concomitant and adjuvant temozolomide for glioblastoma. N Engl J Med 352, 987–996. 15758009 10.1056/NEJMoa043330

[4] Hambardzumyan D and Bergers G (2015) Glioblastoma: defining tumor niches. Trends Cancer 1, 252–265. 27088132 10.1016/j.trecan.2015.10.009 PMC4831073

[5] Ou A , Yung WKA and Majd N (2020) Molecular mechanisms of treatment resistance in glioblastoma. Int J Mol Sci 22, 351. 33396284 10.3390/ijms22010351 PMC7794986

[6] Ceccarelli M , Barthel FP , Malta TM , Sabedot TS , Salama SR , Murray BA , Morozova O , Newton Y , Radenbaugh A , Pagnotta SM *et al*. (2016) Molecular profiling reveals biologically discrete subsets and pathways of progression in diffuse glioma. Cell 164, 550–563. 26824661 10.1016/j.cell.2015.12.028 PMC4754110

[7] Saadeh FS , Mahfouz R and Assi HI (2018) EGFR as a clinical marker in glioblastomas and other gliomas. Int J Biol Markers 33, 22–32. 28885661 10.5301/ijbm.5000301

[8] Gan HK , Cvrljevic AN and Johns TG (2013) The epidermal growth factor receptor variant III (EGFRvIII): where wild things are altered. FEBS J 280, 5350–5370. 23777544 10.1111/febs.12393

[9] Pascale RM , Calvisi DF , Simile MM , Feo CF and Feo F (2020) The Warburg effect 97 years after its discovery. Cancers (Basel) 12, 2819. 33008042 10.3390/cancers12102819 PMC7599761

[10] Strickland M and Stoll EA (2017) Metabolic reprogramming in glioma. Front Cell Dev Biol 5, 43. 28491867 10.3389/fcell.2017.00043 PMC5405080

[11] Cantor JR and Sabatini DM (2012) Cancer cell metabolism: one hallmark, many faces. Cancer Discov 2, 881–898. 23009760 10.1158/2159-8290.CD-12-0345 PMC3491070

[12] Zahra K , Dey T , Ashish , Mishra SP and Pandey U (2020) Pyruvate kinase M2 and cancer: the role of PKM2 in promoting tumorigenesis. Front Oncol 10, 159. 32195169 10.3389/fonc.2020.00159 PMC7061896

[13] Desai S , Ding M , Wang B , Lu Z , Zhao Q , Shaw K , Yung WK , Weinstein JN , Tan M and Yao J (2014) Tissue‐specific isoform switch and DNA hypomethylation of the pyruvate kinase PKM gene in human cancers. Oncotarget 5, 8202–8210. 24077665 10.18632/oncotarget.1159 PMC4226677

[14] Zhou Z , Li M , Zhang L , Zhao H , Şahin Ö , Chen J , Zhao JJ , Songyang Z and Yu D (2018) Oncogenic kinase‐induced PKM2 tyrosine 105 phosphorylation converts nononcogenic PKM2 to a tumor promoter and induces cancer stem‐like cells. Cancer Res 78, 2248–2261. 29440169 10.1158/0008-5472.CAN-17-2726 PMC5932213

[15] Yang W , Zheng Y , Xia Y , Ji H , Chen X , Guo F , Lyssiotis CA , Aldape K , Cantley LC and Lu Z (2012) ERK1/2‐dependent phosphorylation and nuclear translocation of PKM2 promotes the Warburg effect. Nat Cell Biol 14, 1295–1304. 23178880 10.1038/ncb2629 PMC3511602

[16] Yang W , Xia Y , Cao Y , Zheng Y , Bu W , Zhang L , You MJ , Koh MY , Cote G , Aldape K *et al*. (2012) EGFR‐induced and PKCε monoubiquitylation‐dependent NF‐κB activation upregulates PKM2 expression and promotes tumorigenesis [published correction appears in Mol Cell. Jan 18;69(2):347]. Mol Cell 48, 771–784. 23123196 10.1016/j.molcel.2012.09.028 PMC3526114

[17] Laezza C , Notarnicola M , Caruso MG , Messa C , Macchia M , Bertini S , Minutolo F , Portella G , Fiorentino L , Stingo S *et al*. (2006) N6‐isopentenyladenosine arrests tumor cell proliferation by inhibiting farnesyl diphosphate synthase and protein prenylation. FASEB J 20, 412–418. 16507758 10.1096/fj.05-4044lsf

[18] Pisanti S , Picardi P , Ciaglia E , Margarucci L , Ronca R , Giacomini A , Malfitano AM , Casapullo A , Laezza C , Gazzerro P *et al*. (2014) Antiangiogenic effects of N6‐isopentenyladenosine, an endogenous isoprenoid end product, mediated by AMPK activation. FASEB J 28, 1132–1144. 24265487 10.1096/fj.13-238238

[19] Ciaglia E , Pisanti S , Picardi P , Laezza C , Sosa S , Tubaro A , Vitale M , Gazzerro P , Malfitano AM and Bifulco M (2014) N6‐isopentenyladenosine affects cytotoxic activity and cytokines production by IL‐2 activated NK cells and exerts topical anti‐inflammatory activity in mice. Pharmacol Res 89, 1–10. 25063359 10.1016/j.phrs.2014.07.003

[20] Navarra G , Pagano C , Pacelli R , Crescenzi E , Longobardi E , Gazzerro P , Fiore D , Pastorino O , Pentimalli F , Laezza C *et al*. (2020) N6‐isopentenyladenosine enhances the radiosensitivity of glioblastoma cells by inhibiting the homologous recombination repair protein RAD51 expression. Front Oncol 9, 1498. 31993371 10.3389/fonc.2019.01498 PMC6971108

[21] Ciaglia E , Abate M , Laezza C , Pisanti S , Vitale M , Seneca V , Torelli G , Franceschelli S , Catapano G , Gazzerro P *et al*. (2017) Antiglioma effects of N6‐isopentenyladenosine, an endogenous isoprenoid end product, through the downregulation of epidermal growth factor receptor. Int J Cancer 140, 959–972. 27813087 10.1002/ijc.30505

[22] Pagano C , Navarra G , Pastorino O , Avilia G , Coppola L , Della Monica R , Chiariotti L , Florio T , Corsaro A , Torelli G *et al*. (2021) N6‐isopentenyladenosine hinders the vasculogenic mimicry in human glioblastoma cells through Src‐120 catenin pathway modulation and RhoA activity inhibition. Int J Mol Sci 22, 10530. 34638872 10.3390/ijms221910530 PMC8508824

[23] Pagano C , Navarra G , Coppola L , Avilia G , Pastorino O , Della Monica R , Buonaiuto M , Torelli G , Caiazzo P , Bifulco M *et al*. (2022) N6‐isopentenyladenosine induces cell death through necroptosis in human glioblastoma cells. Cell Death Discov 8, 173. 35393392 10.1038/s41420-022-00974-x PMC8991250

[24] Pagano C , Coppola L , Navarra G , Avilia G , Bruzzaniti S , Piemonte E , Galgani M , Della Monica R , Chiariotti L , Cuomo M *et al*. (2022) N6‐Isopentenyladenosine impairs mitochondrial metabolism through inhibition of EGFR translocation on mitochondria in glioblastoma cells. Cancers (Basel) 14, 6044. 36551529 10.3390/cancers14246044 PMC9776489

[25] Thorne AH , Zanca C and Furnari F (2016) Epidermal growth factor receptor targeting and challenges in glioblastoma. Neuro Oncol 18, 914–918. 26755074 10.1093/neuonc/nov319 PMC4896544

[26] Carbone F , Bruzzaniti S , Fusco C , Colamatteo A , Micillo T , De Candia P , Bonacina F , Norata GD and Matarese G (2021) Metabolomics, lipidomics, and immunometabolism. Methods Mol Biol 2285, 319–328. 33928562 10.1007/978-1-0716-1311-5_24

[27] Boschert V , Teusch J , Müller‐Richter UDA , Brands RC and Hartmann S (2022) PKM2 modulation in head and neck squamous cell carcinoma. Int J Mol Sci 23, 775. 35054968 10.3390/ijms23020775 PMC8775697

[28] Ghosh S and Hayden MS (2008) New regulators of NF‐kappaB in inflammation. Nat Rev Immunol 8, 837–848. 18927578 10.1038/nri2423

[29] Rabinowitz JD and Enerbäck S (2020) Lactate: the ugly duckling of energy metabolism. Nat Metab 2, 566–571. 32694798 10.1038/s42255-020-0243-4 PMC7983055

[30] Almouhanna F , Blagojevic B , Can S , Ghanem A and Wölfl S (2021) Pharmacological activation of pyruvate kinase M2 reprograms glycolysis leading to TXNIP depletion and AMPK activation in breast cancer cells. Cancer Metab 9, 5. 33482908 10.1186/s40170-021-00239-8 PMC7821649

[31] Zhang Z , Deng X , Liu Y , Liu Y , Sun L and Chen F (2019) PKM2, function and expression and regulation. Cell Biosci 9, 52. 31391918 10.1186/s13578-019-0317-8 PMC6595688

[32] Park JH , Lee JS , Oh Y , Lee JS , Park HE , Lee H , Park YS , Kyung SY , Kim HS and Yoon S (2022) PKM2 is overexpressed in glioma tissues, and its inhibition highly increases late apoptosis in U87MG cells with low‐density specificity. In Vivo 36, 694–703. 35241524 10.21873/invivo.12755 PMC8931915

[33] Yang W , Xia Y , Ji H , Zheng Y , Liang J , Huang W , Gao X , Aldape K and Lu Z (2011) Nuclear PKM2 regulates β‐catenin transactivation upon EGFR activation. Nature 480, 118–122. 22056988 10.1038/nature10598 PMC3235705

[34] Yang W , Xia Y , Hawke D , Li X , Liang J , Xing D , Aldape K , Hunter T , Alfred Yung WK and Lu Z (2012) PKM2 phosphorylates histone H3 and promotes gene transcription and tumorigenesis. Cell 150, 685–696. 22901803 10.1016/j.cell.2012.07.018 PMC3431020

[35] Han D , Wei W , Chen X , Zhang Y , Wang Y , Zhang J , Wang X , Yu T , Hu Q , Liu N *et al*. (2015) NF‐κB/RelA‐PKM2 mediates inhibition of glycolysis by fenofibrate in glioblastoma cells. Oncotarget 6, 26119–26128. 26172294 10.18632/oncotarget.4444 PMC4694890

[36] Laezza C , Malfitano AM , Di Matola T , Ricchi P and Bifulco M (2010) Involvement of Akt/NF‐κB pathway in N6‐isopentenyladenosine‐induced apoptosis in human breast cancer cells. Mol Carcinog 49, 892–901. 20672320 10.1002/mc.20666

[37] Binderup T , Knigge UP , Federspiel B , Sommer P , Hasselby JP , Loft A and Kjaer A (2013) Gene expression of glucose transporter 1 (GLUT1), hexokinase 1 and hexokinase 2 in gastroenteropancreatic neuroendocrine tumors: correlation with F‐18‐fluorodeoxyglucose positron emission tomography and cellular proliferation. Diagnostics (Basel) 3, 372–384. 26824929 10.3390/diagnostics3040372 PMC4665527

[38] Han W , Shi J , Cao J , Dong B and Guan W (2020) Emerging roles and therapeutic interventions of aerobic glycolysis in glioma. Onco Targets Ther 13, 6937–6955. 32764985 10.2147/OTT.S260376 PMC7371605

[39] Verma H , Cholia RP , Kaur S , Dhiman M and Mantha AK (2021) A short review on cross‐link between pyruvate kinase (PKM2) and glioblastoma multiforme. Metab Brain Dis 36, 751–765. 33651273 10.1007/s11011-021-00690-y

[40] Dong G , Mao Q , Xia W , Xu Y , Wang J , Xu L and Jiang F (2016) PKM2 and cancer: the function of PKM2 beyond glycolysis. Oncol Lett 11, 1980–1986. 26998110 10.3892/ol.2016.4168 PMC4774429

