# Supplementary figures and images for: N6‐isopentenyladenosine inhibits aerobic glycolysis in glioblastoma cells by targeting PKM2 expression and activity
Source: FEBS Open Bio. 2024 Mar 21;14(5):843–54. doi: 10.1002/2211-5463.13766 (PMC11073503; doi:10.1002/2211-5463.13766)

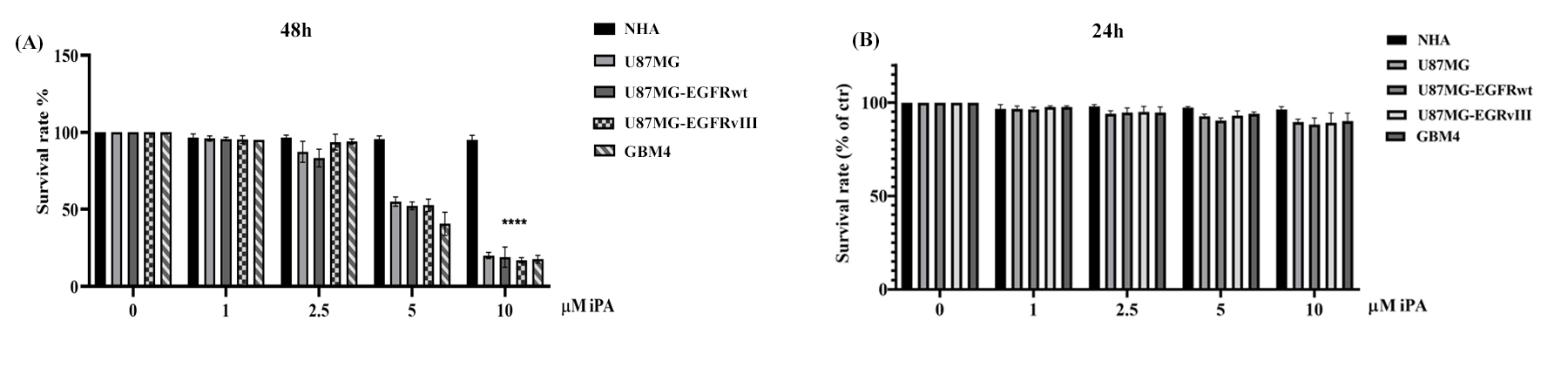

Supplement: Supplementary file 1 — Fig. S1. iPA effects on GBM cells proliferation and viability. (A) BrdU assay showing the effects of scalar doses of iPA (1–10 μm) after 48 h on NHA and GBM cell lines. Notably, iPA did not elicit any effects on the proliferation of NHA cells, the normal counterpart of GBM cells. (B) MTT assay showing that iPA (1–10 μm) after 24 h had no significant effects on GBM cells and NHA viability. All data are presented as the mean ± SD of at least three independent experiments. ****P < 0.0001 (Student's t‐test and one/two‐way ANOVA). [file FEB4-14-843-s004.tif]

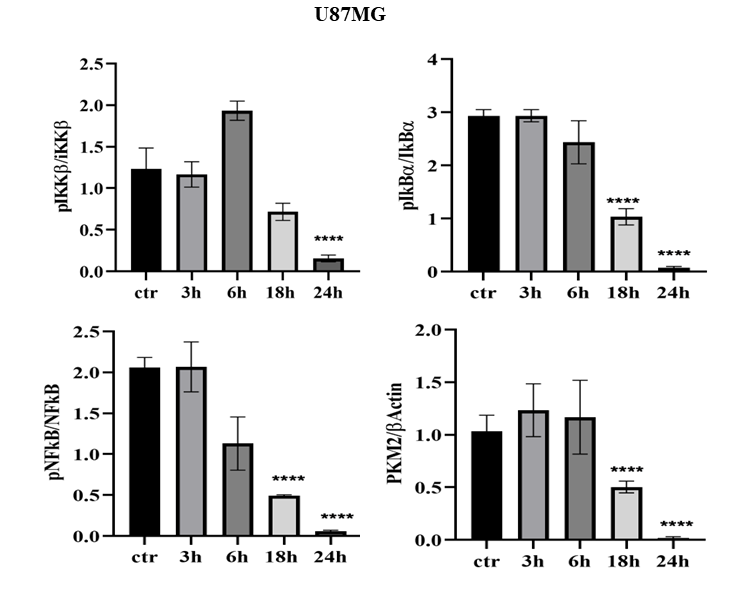

Supplement: Supplementary file 2 — Fig. S2. Western blot densitometry analysis of U87MG. Histograms showing densitometry analysis of p‐IKKβ/IKKβ, p‐IkBα/IkBα, p‐NF‐kB/NF‐kB and PKM2/β‐actin bands of the U87MG cell line. All data are presented as the mean ± SD of at least three independent experiments. ****P < 0.0001 (Student's t‐test and one/two‐way ANOVA). [file FEB4-14-843-s002.tif]

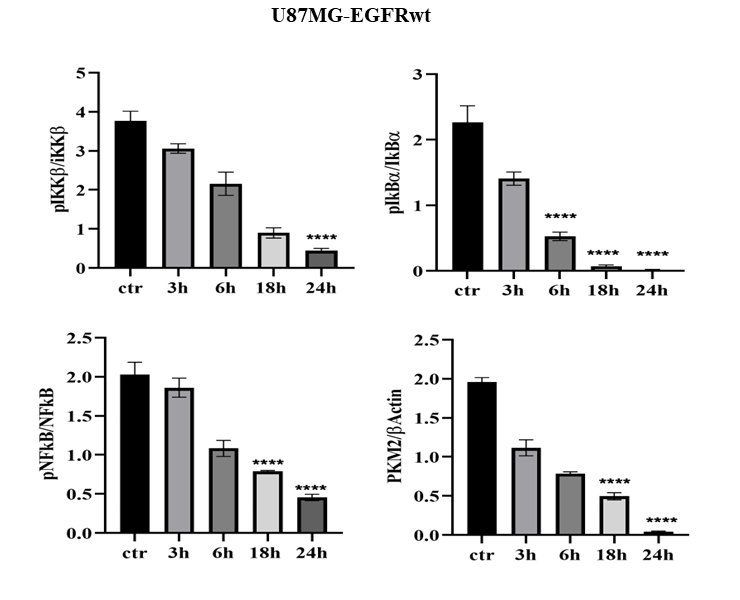

Supplement: Supplementary file 3 — Fig. S3. Western blot densitometry analysis of U87MG‐EGFRwt. Histograms showing densitometry analysis of p‐IKKβ/IKKβ, p‐IkBα/IkBα, p‐NF‐kB/NF‐kB and PKM2/β‐actin bands of the U87MG‐EGFRwt cell line. All data are presented as the mean ± SD of at least three independent experiments. ****P < 0.0001 (Student's t‐test and one/two‐way ANOVA). [file FEB4-14-843-s003.tif]

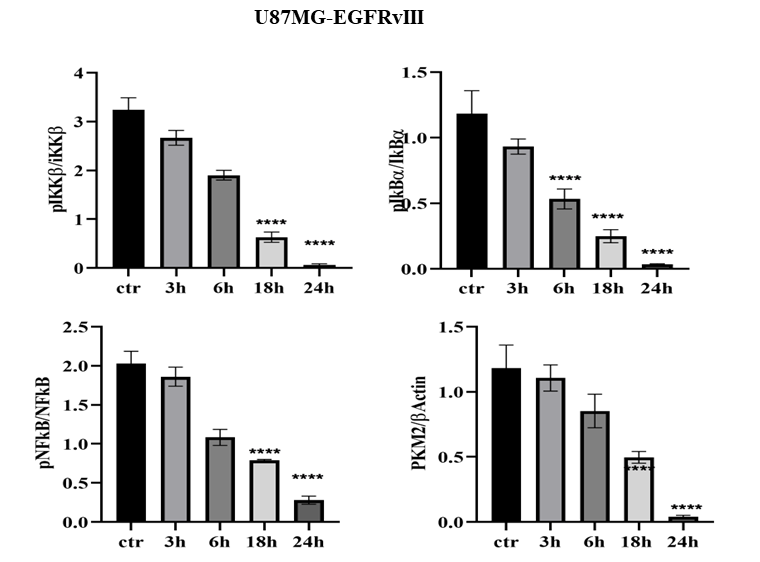

Supplement: Supplementary file 4 — Fig. S4. Western blot densitometry analysis of U87MG‐EGFRvIII. Histograms showing densitometry analysis of p‐IKKβ/IKKβ, p‐IkBα/IkBα, p‐NF‐kB/NF‐kB and PKM2/β‐actin bands of the U87MG‐EGFRvIII cell line. All data are presented as the mean ± SD of at least three independent experiments. ****P < 0.0001 (Student's t‐test and one/two‐way ANOVA). [file FEB4-14-843-s001.tif]

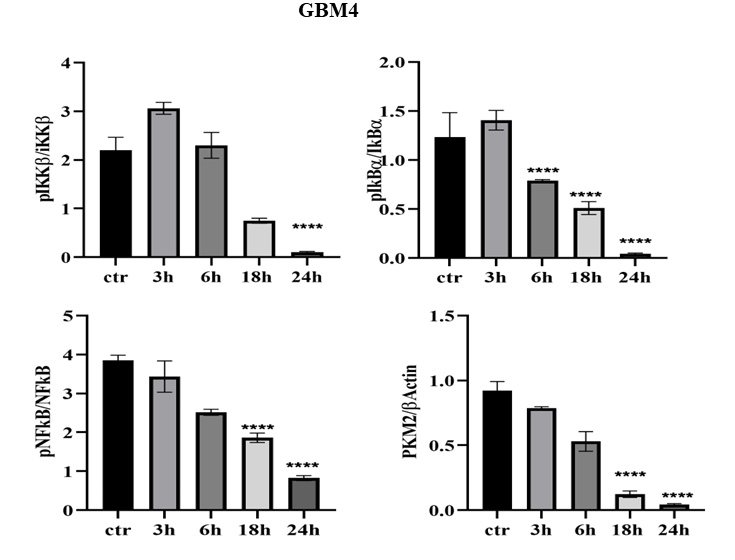

Supplement: Supplementary file 5 — Fig. S5. Western blot densitometry analysis of GBM4. Histograms showing densitometry analysis of p‐IKKβ/IKKβ, p‐IkBα/IkBα, p‐NF‐kB/NF‐kB and PKM2/β‐actin bands of the GBM4 cell line. All data are presented as the mean ± SD of at least three independent experiments. ****P < 0.0001 (Student's t‐test and one/two‐way ANOVA). [file FEB4-14-843-s005.tif]
